# Short-Term Effectiveness of a Mobile Phone App for Increasing Physical Activity and Adherence to the Mediterranean Diet in Primary Care: A Randomized Controlled Trial (EVIDENT II Study)

**DOI:** 10.2196/jmir.6814

**Published:** 2016-12-19

**Authors:** Jose I Recio-Rodriguez, Cristina Agudo-Conde, Carlos Martin-Cantera, Mª Natividad González-Viejo, Mª Del Carmen Fernandez-Alonso, Maria Soledad Arietaleanizbeaskoa, Yolanda Schmolling-Guinovart, Jose-Angel Maderuelo-Fernandez, Emiliano Rodriguez-Sanchez, Manuel A Gomez-Marcos, Luis Garcia-Ortiz, EVIDENT Investigators

**Affiliations:** ^1^Biomedical Research Institute of Salamanca (IBSAL)Primary Health Care Research Unit, La Alamedilla Health CenterCastilla León Health ServiceSalamancaSpain; ^2^University of SalamancaDepartment of Nursing and PhysiotherapySalamancaSpain; ^3^Primary Health Care University Research Institute IDIAP-Jordi GolPasseig de Sant Joan Health Center, Catalan Health ServiceDepartment of Medicine. University Autonoma of BarcelonaBarcelonaSpain; ^4^Torre Ramona Health Center, Aragon Health ServiceZaragozaSpain; ^5^Casa del Barco Health Center, Castilla y León Health ServiceValladolidSpain; ^6^Primary Health Care Research Unit of Bizkaia.Basque Health Service-OsakidetzaBilbaoSpain; ^7^Río Tajo Health Center, Castilla-La Mancha Health ServiceUniversity of Castilla-La ManchaTalavera de la ReinaSpain; ^8^University of SalamancaDepartment of MedicineSalamancaSpain; ^9^University of SalamancaDepartment of Biomedical and Diagnostic SciencesSalamancaSpain; ^10^EVIDENT Investigators. redIAPP: Spanish Research Network for Preventive Activities and Health Promotion in Primary CareSalamancaSpain

**Keywords:** physical activity, food, information and communication technologies, arterial aging

## Abstract

**Background:**

The use of mobile phone apps for improving lifestyles has become generalized in the population, although little is still known about their effectiveness in improving health.

**Objective:**

We evaluate the effect of adding an app to standard counseling on increased physical activity (PA) and adherence to the Mediterranean diet, 3 months after implementation.

**Methods:**

A randomized, multicenter clinical trial was carried out. A total of 833 participants were recruited in six primary care centers in Spain through random sampling: 415 in the app+counseling group and 418 in the counseling only group. Counseling on PA and the Mediterranean diet was given to both groups. The app+counseling participants additionally received training in the use of an app designed to promote PA and the Mediterranean diet over a 3-month period. PA was measured with the 7-day Physical Activity Recall (PAR) questionnaire and an accelerometer; adherence to the Mediterranean diet was assessed using the Mediterranean Diet Adherence Screener questionnaire.

**Results:**

Participants were predominantly female in both the app+counseling (249/415, 60.0%) and counseling only (268/418, 64.1%) groups, with a mean age of 51.4 (SD 12.1) and 52.3 (SD 12.0) years, respectively. Leisure-time moderate-to-vigorous physical activity (MVPA) by 7-day PAR increased in the app+counseling (mean 29, 95% CI 5-53 min/week; *P*=.02) but not in the counseling only group (mean 17.4, 95% CI –18 to 53 min/week; *P*=.38). No differences in increase of activity were found between the two groups. The accelerometer recorded a decrease in PA after 3 months in both groups: MVPA mean –55.3 (95% CI –75.8 to –34.9) min/week in app+counseling group and mean –30.1 (95% CI –51.8 to –8.4) min/week in counseling only group. Adherence to the Mediterranean diet increased in both groups (8.4% in app+counseling and 10.4% in counseling only group), with an increase in score of 0.42 and 0.53 points, respectively (*P*<.001), but no difference between groups (*P*=.86).

**Conclusions:**

Leisure-time MVPA increased more in the app+counseling than counseling only group, although no difference was found when comparing the increase between the two groups. Counseling accompanied by printed materials appears to be effective in improving adherence to the Mediterranean diet, although the app does not increase adherence.

**ClinicalTrial:**

Clinicaltrials.gov NCT02016014; https://clinicaltrials.gov/ct2/show/NCT02016014 (Archived by WebCite at http://www.webcitation.org/6mnopADbf)

## Introduction

Regular physical activity (PA) offers considerable physical and psychological health benefits [1], and reduces overall and cardiovascular mortality [2,3] in the general population [4,5]. Despite this fact, most of the population in developed countries do not follow the international recommendations on PA [6,7]. In phase 1 of the “Lifestyles and Arterial Aging” (EVIDENT) trial [8], the proportion of active individuals was very low (31%). Interventions designed to promote PA have revealed a small to moderate effect [9], and could be useful for increasing the number of people adhering to recommended levels of PA. The Mediterranean diet has been shown to be effective in preventing cardiovascular disease [10], and other disorders such as type 2 diabetes [11]. Adherence to the Mediterranean diet is low at only 33%, as documented by the EVIDENT trial [12]. Interventions designed to improve adherence to the Mediterranean diet showed that more frequent contact or intensive interventions appear to be more effective [13].

Information and communication technologies are currently one of the supporting tools that may facilitate such reinforcement and contribute to improving health and changing lifestyles [14]. Many mobile phone apps have been developed with this aim in mind, although the supporting evidence is generally limited [15]. Furthermore, the results are not always uniform, with positive effects in terms of weight loss [16,17], but few or contradictory effects on PA [18,19]. However, few studies have examined effectiveness in large population samples using an app combining PA and food habits.

This study evaluates the short-term (3 months) effects of adding an app in support of standardized counseling to increase PA and adherence to the Mediterranean diet.

## Methods

### Design

A multicenter randomized controlled trial with two parallel groups was carried out with a follow-up period of 12 months (the EVIDENT II study) [20]. Assessments were made at baseline and after 3 months between January 2014 and December 2015, with evaluation at 12 months in 2016.

### Setting and Participants

**Figure 1:**
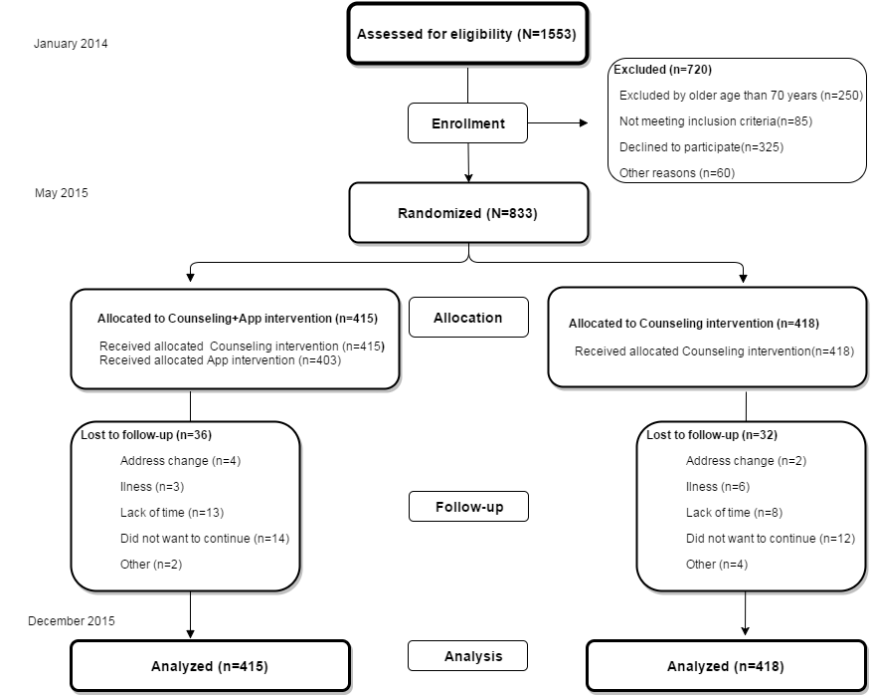
Study flowchart: enrollment of participants and completion of study.

The study population was selected from the EVIDENT I study [21], including 1553 participants randomly selected from six primary care centers in family practice offices. Participants older than 70 years of age were excluded, as were those unable to do exercise or follow the Mediterranean diet, as well as those individuals meeting any of the exclusion criteria of the EVIDENT I study [20]. Of the participants recruited in the EVIDENT I study, 833 were included in this study (Figure 1) and these participants were randomized in a 1:1 proportion on a centralized basis from Salamanca, using the Epidat 4.0 software package to the counseling+app group (n=415) or the counseling only group (n=418). The investigator who performed the data analysis was blinded. Due to the nature of the study, the participants could not be blinded to the intervention.

The study was approved by the Clinical Research Ethics Committee of the health care area of Salamanca (CEIC de Area de Salud de Salamanca, June 21, 2013) as the reference Committee. In addition, the study was approved by the Ethics Committees of the five collaborating centers (CEIC de Aragón [CEICA], CEIC de IDIAP Jordi Gol, CEIC de Euskadi [CEIC-E], CEIC de Castilla la Mancha, and CEIC de Area de Salud de Valladolid Oeste). All participants signed the informed consent form prior to inclusion in the study, in accordance with the Declaration of Helsinki [22].

### Intervention

A research nurse performed a common intervention lasting 30 minutes in both groups. The intervention consisted of standardized counseling in PA and the Mediterranean diet, with the delivery of printed supporting material (leaflet) on the session. The effectiveness of these interventions has been demonstrated in the “Experimental Program for Physical Activity Promotion” (PEPAF) study [23] and the “Prevención con Dieta Mediterránea” (PREDIMED) study [10].

For the counseling+app group *,* the participants also received training in the use of a mobile phone app designed to promote the Mediterranean diet and increase PA over a 3-month period. The app was designed by software engineers in collaboration with dietitians and PA experts, with an easy-to-use interface for logging food and exercise. A 15-minute initial visit was used to provide training in the use of the device, which was employed daily for the full 3-month period of the intervention. The participants were instructed to enter their food intake (breakfast, lunch, afternoon snack, and dinner). Based on adequate proportions of macronutrients (carbohydrates: 50%-60%; protein: 10%-15%; fats: 30%-35%; fiber: >22 g/day), following recommendations of the of the , National Academy of Sciences (US Department of Health and Human Services), a personalized recommendation was provided. Regular PA was recorded with the accelerometer of the device, together with user input of activities performed without the mobile phone (eg, swimming, football). Lastly, at the end of the day, the app reported a summary of food intake and PA performed and a balance of ingested and spent calories. The app, in turn, generated a recommended plan for the following days, with a view to improve eating habits and increase PA. A new visit took place 1 week after supplying the device to confirm that it was being used correctly. The mobile phone was returned after 3 months, coinciding with the common follow-up visit. Adherence to the mobile phone app was assessed by the number of days of recordings in the device.

### Outcome Measures and Follow-Up

The main outcome measures were change in PA and adherence to the Mediterranean diet at 3 months in the app+counseling group compared to the counseling only group. Other outcome measures were also collected, including blood pressure, waist circumference, body mass index (BMI), and laboratory parameters. A detailed description has been published elsewhere of how clinical data were collected [20].

#### Physical Activity

Physical activity was measured with an accelerometer and the 7-day Physical Activity Recall (7-day PAR) questionnaire. The ActiGraph GT3X accelerometer (ActiGraph, Shalimar, FL, USA) was used and has been previously validated [24-26]. Activity “counts” were recorded to the internal memory of the accelerometers by converting acceleration units over a given epoch [27]. Participants wore the accelerometer fastened with an elastic strap to the right side of the waist for seven consecutive days during habitual PA, except for bathing and performing activities in the water. The accelerometer was set to record PA data every minute. Inclusion criteria were a minimum of 5 days of recording, including at least one weekend day and at least 600 registered minutes per day. The first and last day’s data were excluded to analyze full days only, and the uptime was adjusted to 7 days. The main outcome variable from the activity monitor was the mean intensity of PA (counts/minute). The intensity of PA was rated according to the cut-off points proposed by Freedson [28].

The 7-day PAR is a semistructured interview (10-15 minutes) in which participants provide an estimate of the number of hours dedicated to physical or occupational activities requiring at least a moderate effort in the past 7 days. The categories collected are moderate, vigorous, and very vigorous PA. The amount of time dedicated to each activity was multiplied by the mean metabolic equivalent (MET) of each category: light activity=1.5, moderate activity=4, vigorous activity=6, and very vigorous activity=10. The dose of physical exercise was estimated in MET-minutes/week. Active individuals were considered as those doing at least 30 minutes of moderate activity 5 days per week, or at least 20 minutes of vigorous activity 3 days per week [29].

#### Nutrition

Adherence to the Mediterranean diet, as a nutrition primary endpoint, was measured using the validated 14-point Mediterranean Diet Adherence Screener (MEDAS) [30], developed by the PREDIMED study group. The 14-item screener includes 12 questions on food consumption frequency and two questions on food intake habits considered characteristic of the Spanish Mediterranean diet. Each question was scored as 0 or 1, and the total score ranged from 0 to 14. Adequate adherence to the Mediterranean diet was assumed when the total score was ≥9 points.

### Statistical Analysis

Estimation of sample size was made for the main study endpoints. Assuming alpha=.05 and beta=.20, with a SD 154 counts/minute, we would need 828 participants (414 per group) to detect an increase of 30 counts/minute in the app+counseling versus counseling only group; for a SD of 2 points in Mediterranean diet, we would need 504 participants (252 per group) to detect an increase of 0.5 points. We considered it sufficient to include 833 participants in order to detect clinically relevant differences in the main study endpoints.

The results were expressed as mean and standard deviation for quantitative variables and as the frequency distribution for qualitative variables. Analysis of the results was made on an intention-to-treat basis. The chi-square test and Fisher test were used to analyze the association between independent qualitative variables. The Student *t* test was used for the comparison of means between two groups and the paired *t* test was applied to assess changes within the same group. Analysis of variance (ANOVA) was used for comparison of means between more than two groups. In order to analyze the effect of the intervention, we compared the changes observed between the counseling only and app+counseling groups by ANCOVA test, adjusting for baseline measures of each variable. To evaluate the effect of adherence to the app in the increase of PA measured by the accelerometer, we performed a multivariate analysis based on the general lineal model, adjusting the results for age and sex. The intraclass correlation coefficients of outcome data for centers were for MET-minutes/week in leisure time (7 day PAR; ρ=.009), for total moderate-to-vigorous PA (MVPA) time/week (accelerometer; ρ=.002), and for the score of adherence to Mediterranean diet (MEDAS; ρ=.009). The contrasting of hypotheses established alpha=.05. The data were analyzed using SPSS version 23.0 (IBM Corp, Armonk, NY, USA). A value of *P*<.05 was considered statistically significant.

## Results

### Baseline Characteristics of the Participants and Follow-Up

The 833 participants were predominantly females in both the app+counseling (249/415, 60.0%) and counseling only (268/418, 64.1%) groups, with a mean age of 51.4 (SD 12.1) and 52.3 (SD 12.0) years, respectively. Likewise, no differences were observed between the two groups in terms of the other demographic and clinical characteristics (Table 1). Regarding PA evaluated with the 7-day PAR, we found no significant difference between the groups; app+counseling reached a mean 864.6 (SD 1407.7) MET-minutes/week, whereas the counseling only group reached a mean 865.8 (SD 1330.6) MET-minutes/week (*P*=.34). For the parameters analyzed with the accelerometer, the results were also similar in both groups (Table 2). For adherence to the Mediterranean diet, the mean score was 7.6 (SD 2.1) in the app+counseling group and mean 7.4 (SD 1.9) in the counseling only group, with an adequate adherence rate of 34.2% (142/315) in the app+counseling group versus 28.5% (119/318) in the counseling only group (*P*=.09) (Table 2 and Multimedia Appendix 1).

**Table 1:** Baseline characteristics of the study population (N=833).

Variable		App+counseling (n=415)	Counseling only (n=418)	*P* value
Age (years), mean (SD)		51.4 (12.1)	52.3 (12.0)	.28
Gender (female), n (%)		249 (60.0)	268 (64.1)	.23
**Work situation, n (%)**				.25
	Works outside home	228 (54.9)	203 (48.6)	
	Homemaker	53 (12.8)	72 (17.2)	
	Retired	77 (18.6)	89 (21.3)	
	Student	10 (2.4)	8 (1.9)	
	Unemployed	47 (11.3)	46 (11.0)	
**Educational level, n (%)**				
	University studies	117 (28.2)	132 (31.6)	.42
	Middle or high school	208 (50.1)	208 (49.8)	
	Elementary school	90 (21.7)	78 (18.7)	
**Smoking, n (%)**				.20
	Nonsmoker	190 (45.8)	166 (39.7)	
	Smoker	94 (22.7)	108 (25.8)	
	Former smoker	131 (31.6)	144 (34.4)	
Waist circumference (cm), mean (SD)		95.2 (13.2)	94.8 (13.1)	.71
BMI (kg/m^2^), mean (SD)		28.1 (5.1)	27.6 (4.59)	.14
**BMI category, n (%)**				.50
	<25	117 (28.2)	131 (31.3)	
	25-30	172 (41.4)	173 (41.4)	
	>30	126 (30.4)	114 (27.3)	
Systolic blood pressure (mmHg), mean (SD)		124 (16)	124 (17)	.75
Diastolic blood pressure (mmHg), mean (SD)		76 (10)	76 (10)	.41
Total cholesterol (mg/dL), mean (SD)		202 (35)	206 (37)	.08
Triglycerides (mg/dL), mean (SD)		112 (63)	107 (67)	.29
Glycated hemoglobin (%), mean (SD)		5.5 (0.5)	5.5 (0.6)	.87
Hypertension, n (%)		145 (34.9)	133 (31.8)	.34
Dyslipidemia, n (%)		116 (28.2)	113 (27.3)	.77
Diabetes, n (%)		32 (7.7)	30 (7.2)	.77
**Medication use, n (%)**				
	Antihypertensive drugs	108 (26.0)	95 (22.7)	.29
	Lipid-lowering drugs	90 (21.7)	74 (17.7)	.16
	Antidiabetic drugs	24 (5.8)	28 (6.7)	.67
**Physical activity stage of change, n (%)^a^**				.35
	Precontemplation	57 (14.0)	73 (17.7)	
	Contemplation	28 (6.9)	38 (9.2)	
	Preparation	58 (14.2)	51 (12.4)	
	Action	30 (7.4)	26 (6.3)	
	Maintenance	235 (57.6)	224 (54.4)	
**Dietary habits stage of change, n (%)**				.91
	Precontemplation	34 (8.3)	34 (8.2)
	Contemplation	26 (6.3)	20 (4.8)	
	Preparation	59 (14.3)	57 (13.8)	
	Action	18 (4.4)	18 (4.3)	
	Maintenance	275 (66.7)	285 (68.8)	
Participants with total Mediterranean diet score ≥9 points, n (%)		142 (34.2)	119 (28.5)	.09
Score for adherence to Mediterranean diet, mean (SD)		7.6 (2.1)	7.4 (2.0)	.09

^a^ Stage of change by Prochaska and Diclemente model.

**Table 2:** Baseline physical activity by 7-day Physical Activity Recall (PAR) questionnaire and accelerometer data (N=833).

Measures of physical activity		App+counseling (n=415)	Counseling only (n=418)	*P* value
**7-day PAR^a^**				
	Total minutes moderate activity, mean (SD)	152.7 (264.8)	154.9 (258.2)	.90
	Total minutes moderate activity in leisure time, mean (SD)	131.2 (213.1)	148.0 (249.4)	.29
	Total minutes vigorous/very vigorous activity, mean (SD)	29.9 (99.2)	30.0 (106.2)	.98
	Total minutes vigorous/very vigorous activity in leisure time, mean (SD)	28.0 (97.1)	27.9 (98.2)	.99
	Total minutes moderate vigorous/very vigorous activity, mean (SD)	182.6 (293.0)	184.9 (284.7)	.91
	Total minutes moderate vigorous/very vigorous activity in leisure time, mean (SD)	159.1 (228.9)	175.9 (271.6)	.33
	MET-minutes/week, mean (SD)	864.6 (1407.8)	865.8 (1330.6)	.99
	MET-minutes/week in leisure time, mean (SD)	764.4 (1119.7)	825.6 (1263.3)	.46
	Active,^b^ n (%)	114 (27.5)	118 (28.2)	.82
**Accelerometer**				
	Steps/day, mean (SD)	9992.3 (3847.3)	9708.3 (3930.9)	.31
	Counts minute/week, mean (SD)	69.0 (70.4)	65.9 (69.4)	.54
	Sedentary minutes/week, mean (SD)	8327.0 (540.4)	8341.4 (526.0)	.71
	Light minutes/week, mean (SD)	1298.3 (436.9)	1307.2 (423.2)	.77
	Moderate minutes/week, mean (SD)	438.0 (205.4)	413.3 (212.6)	.10
	Vigorous/very vigorous minutes/week, mean (SD)	16.7 (38.9)	18.2 (45.8)	.62
	Total MVPA^c^ minutes/week, mean (SD)	455.4 (215.9)	432.7 (222.5)	.15
	MET-minutes/week, mean (SD)	1850.8 (891.7)	1762.6 (922.0)	.18
	>450 MET-minutes/week, n (%)	368 (96.6)	373 (94.4)	.15

^a^ MET: metabolic equivalent

^b^Active were considered as those doing at least 30 minutes of moderate activity for 5 days a week, or at least 20 minutes of vigorous activity for 3 days a week.

^c^MVPA: moderate-to-vigorous physical activity.

Of the 833 participants included in the study, 36 of 415 (8.6%) in the app+counseling group were lost at the 3-month time point versus 32 of 418 (7.6%) in the counseling only group (Figure 1).

### Changes in Physical Activity and Adherence to the Mediterranean Diet

Based on data from the 7-day PAR, both groups increased their PA after 3 months, although only the app+counseling group reached statistical significance for the criteria leisure-time moderate activity (mean 28, 95% CI 6-50 minutes/week) and leisure-time MVPA (mean 29, 95% CI 5-53 minutes/week). Although the increase in activity in the app+counseling group was greater than in the counseling only group for all analyzed variables, no significant differences were observed between the two groups (Table 3). In relation to PA evaluated with the accelerometer, we recorded a decrease in daily steps, counts/minute, and time spent at the different levels of activity (except for vigorous/very vigorous activity) with an increase in sedentary time in both groups. Furthermore, no differences were observed when comparing the changes between the two groups (Table 2). Both groups increased adherence to the Mediterranean diet to a similar degree after 3 months versus baseline (app+counseling: 8.4%; counseling only: 10.4%), with an increase in overall score of mean 0.42 (95% CI 0.24-0.60) points in the app+counseling group and mean 0.53 (95% CI 0.35-0.71) points in the counseling only group (Table 4).

**Table 3:** Changes in physical activity and sedentary lifestyle at 3 months compared to baseline.

Measures of physical activity		App+counseling (n=379)		Counseling group (n=386)		Mean difference (app+counseling—counseling group)^d^	
		Mean (95% CI)	*P*	Mean (95% CI)	*P*	Mean (95% CI)	*P*
7-day PAR^a^							
	Total minutes moderate activity/week	20.6 (–8.1, 49.2)	.16	8.3 (–18.6, 35.2)	.54	10.7 (–23.8, 45.2)	.54
	Minutes moderate activity in leisure minutes/week	28.4 (6.0, 50.8)	.01	12.8 (–13.2, 38.8)	.33	7.8 (–23.5, 39.2)	.62
	Total minutes vigorous/very vigorous activity/week	2.8 (–7.6, 13.1)	.60	–0.7 (–10.0, 8.7)	.89	3.5 (–8.3, 15.2)	.56
	Minutes vigorous/very vigorous activity in leisure minutes/week	0.7 (–9.5, 10.9)	.89	–1.1 (–10.3, 8.1)	.82	1.9 (–9.1, 13.0)	.73
	Total minutes MVPA^c^/week	23.3 (–5.4, 52.1)	.11	7.7 (–19.8, 35.2)	.58	14.2 (–20.1, 48.5)	.42
	Minutes MVPA in leisure minutes/week	29.1 (4.9, 53.3)	.02	11.7 (–14.6, 38.1)	.38	9.5 (–22.6, 41.6)	.56
	MET^b^-minutes/week	88.8 (–42.8, 220.3)	.18	14.5 (–108.7, 137.8)	.82	72.4 (–76.4, 221.2)	.34
	MET-minutes/week in leisure time	110.5 (–5.0, 225.9)	.06	26.9 (–90.3, 144.2)	.65	51.5 (–90.0, 192.9)	.47
Accelerometer^e^							
	Steps/day	–1042.1 (–1401.7, –682.6)	<.001	–584.2 (–961.2, –207.1)	.002	–354.6 (–833.7, 124.4)	.15
	Counts minutes/week	–12.9 (–18.6, –7.3)	<.001	–6.8 (–13.3, –0.3)	.04	–5.1 (–12.7, 2.5)	.19
	Sedentary minutes/week	167.7 (114.9, 220.5)	<.001	125.6 (73.7, 177.6)	<.001	32.9 (–37.6, 103.3)	.36
	Light minutes/week	–113.0 –(154.4, –71.6)	<.001	–96.6 (–137.3, –55.8)	<.001	–14.3 (–69.6, 41.1)	.61
	Moderate minute /week	–51.3 (–71.3, –31.4)	<.001	–26.3 (–47.0, –5.5)	.01	–14.8 (–40.8, 11.1)	.26
	Vigorous very vigorous minutes/week	–3.4 (–6.9, , 0.2)	.06	–2.8 (–6.7, 1.0)	.15	–1.1 (–5.6, 3.4)	.62
	Total MVPA minutes/week	–55.3 (–75.8, –34.9)	<.001	–30.1 (–51.8, –8.4)	.01	–15.9 (–42.8, 10.9)	.24
	MET-minutes/week	–229.3 (–313.2, –145.4)	<.001	–118.6 (–208.6, –28.7)	.01	74.9 (–186.1, 36.2)	.19

^a^ 7-day PAR:7-day Physical Activity Recall;

^b^MET: metabolic equivalent

^c^MVPA: moderate-to-vigorous physical activity.

^d^ Changes in app+counseling and counseling only group=data at 3 months–baseline.

^e^ In accelerometer measurement, 335 participants in app+counseling group and 344 in counseling only group.

**Table 4:** Changes in the Mediterranean diet at 3 months compared to baseline.

Criteria Mediterranean diet		App+counseling^a^ (n=379)		Counseling group^a^ (n=386)		Mean difference (app+counseling–counseling)	
		% Mean (95% CI)	*P*	% Mean (95% CI)	*P*	% Mean (95% CI)	*P*
Using olive oil as the principal source of fat for cooking		3.2 (0.9, 5.5)	.01	2.6 (0.4, 4.8)	.02	0.6 (–2.6, 3.7)	.72
≥4 tbsp (54 g) of olive oil/day (eg, used in frying, salads, meals eaten away from home)		0.5 (–4.4, 5.5)	.83	2.1 (–2.8, 6.9)	.40	–1.6 (–8.5, 5.4)	.66
<.0017.6 (2.9, 12.2).0010.4 (–6.0, 6.8).902 or more servings of vegetables/day		8.2 (3.1, 13.3)	.002	12.3 (7.2, 17.4)	<.001	–4.1 (–11.3, 3.2)	.27
1 serving of red meat or sausage/day8.0 (3.5, 12.4) 3 or more pieces of fruit/day		1.6 (–2.6, 5.8)	.45	3.6 (0.1, 7.2)	.04	–2.1 (–7.5, 3.4)	.46
1 serving of animal fat/day		0.8 (–2.2, 3.8)	.60	0.8 (–2.1, 3.7)	.59	0.0 (–4.1, 4.1)	.99
1 cup (100 mL) of sugar-sweetened beverages/day		2.1 (–1.2, 5.4)	.21	1.3 (–2.1, 4.8)	.46	0.8 (–4.0, 5.6)	.74
≥7 servings of red wine/week		–0.3 (–3.4, 2.9)	.87	0.3 (–2.1, 2.6)	.83	–0.5 (–4.5, 3.4)	.79
≥3 servings of legumes/week		–3.4 (–8.1, 1.2)	.14	–1.6 (–5.5, 2.3)	.43	–1.9 (–7.9, 4.1)	.54
≥3 servings of fish/week		5.3 (0.5, 10.1)	.03	3.9 (–0.8, 8.6)	.10 1.4 (–5.3, 8.1)	.68	
<2 commercial pastries/week		6.9 (1.5, 12.2)	.01	8.6 (3.6, 13.7)	.001	–1.7 (–9.1, 5.6)	.64
≥3 servings of nuts/week		2.1 (–2.9, 7.1)	.40	4.7 (–0.2, 9.5)	.05	–2.6 (–9.5, 4.4)	.47
Preferring white meat over red meat?		6.4 (1.4, 11.3)	.01	10.4 (6.3, 14.6)	<.001	–4.1 (–10.5, 2.4)	.22
≥2 servings/week of a dish with a traditional sauce of tomatoes, garlic, onion, or leeks sautéed in olive oil		3.4 (–1.8, 8.7)	.20	–0.5 (–5.9, 4.9)	.85	4.0 (–3.6, 11.5)	.30
Study participants with a total score ≥9 points		8.4 (3.4, 13.5)	.001	10.4 (5.0, 15.8)	<.001	–1.9 (–9.3, 5.5)	.61
Difference Mediterranean diet score		0.42 (0.24, 0.60)	<.001	0.53 (0.35, 0.71)	<.001	–0.02 (–0.25, 0.21)	.86

^a^ Changes in app+counseling and counseling only group=data at 3 months–baseline.

### Adherence to the Mobile Phone App

**Figure 2:**
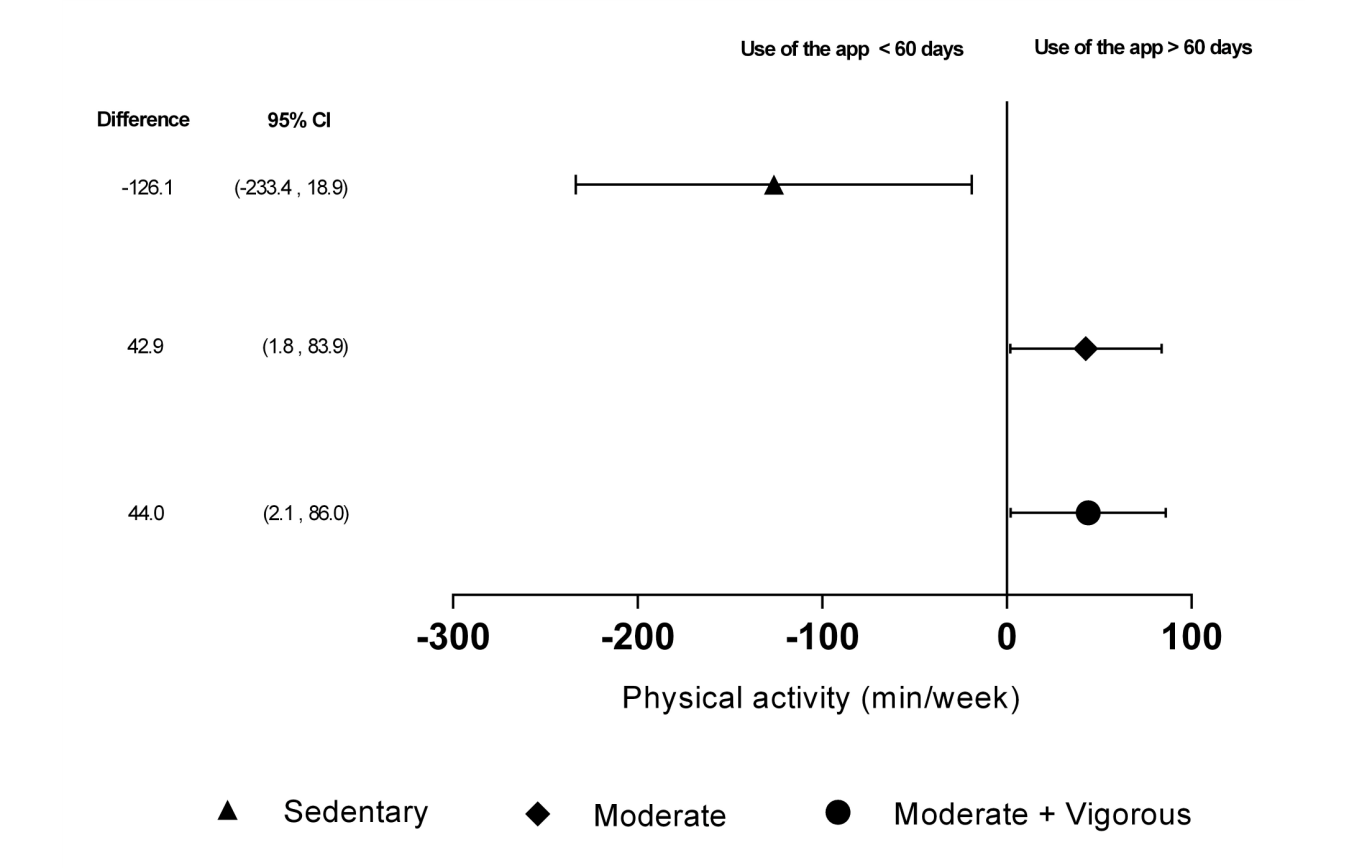
Adherence to the mobile phone app. Changes in physical activity evaluated with the accelerometer, according to adherence to the mobile phone app (number of days with a record in the app). Higher adherence (>60 days): 56.8%, 236/415; lower adherence (≤60 days): 43.1%, 179/415. (0-6 days: 17.8%, 74/415; 7-30 days: 10.3%, 43/415; 31-60 days: 14.9%, 62/415).

In the app+counseling group, 56.8% (236/315) of participants used the mobile phone for more than 60 days. Although there was a decrease in PA after 3 months, as evaluated by the accelerometer in both groups, the decrease was less pronounced in the group that used the mobile phone most (>60 days). This group showed a net increase in the time of moderate PA of mean 42.9 (95%CI 1.8-83.9) minutes/week and MVPA of mean 44.0 (95% CI 2.1-86.0) minutes/week, and a net decrease in sedentary time of mean 126.1 (95% CI 18.9-233.4) minutes/week. We also recorded a net increase in counts/minute of mean 766.6 (95% CI 26.2-1506.9) (Figure 2).

### Analysis by Subgroups

In the male subgroup, we observed a greater decrease in MVPA by accelerometry in the app+counseling than counseling only group (Multimedia Appendixes 2 and 3). In the female subgroup, we observed an increase in leisure-time moderate physical activity as evaluated with the 7-day PAR in the app+counseling versus counseling only group, although the statistical significance was lost after adjusting for baseline data (Multimedia Appendix 4).

## Discussion

### Principal Findings

Although some randomized controlled clinical trials have analyzed the effect of mobile phone apps in promoting healthy lifestyles, the EVIDENT II trial has included the largest number of participants (N=833) and also has the longest follow-up (12 months). The main findings at short-term follow-up (3 months) were an increase in PA as evaluated by the 7-day PAR in both groups (although greater in app+counseling), and an increase in the time dedicated to leisure-time moderate/vigorous activities. However, assessment with the accelerometer revealed a similar decrease in PA in both groups. Adherence to the Mediterranean diet was seen to increase in both groups, as evidenced by the MEDAS score. Lastly, in the accelerometer analysis, the participants in the app+counseling group that most used the app showed a net increase in MVPA time and a net decrease in sedentary time.

### Comparison With Prior Work

At this time, there is still no conclusive evidence of the effectiveness of apps for mobile phones in improving lifestyles. In this regard, the meta-analysis published by Flores et al [16] found that interventions with apps had some impact in terms of weight loss (mean BMI 0.43 kg/m^2^), although no improvement in terms of increased PA was observed. Partridge et al [31], in a sample of 250 participants between ages 18 and 35 years, evaluated PA using the International Physical Activity Questionnaire (IPAQ), with results similar to those obtained in our study with the 7-day PAR. In both cases, there was a greater increase in PA in the intervention group versus the control group, although the differences between them were not significant. In turn, Laing et al [17], in a randomized controlled study of 212 overweight individuals with a mean age of 43 years, found the use of an app (MyFitnessPal) had no impact on either weight loss or increased PA as assessed by questionnaire.

However, the SMART MOVE study [32], which had 90 participants (45 in each group), PA was assessed from the steps estimated by the mobile phone pedometer and an increase was recorded after 8 weeks in the intervention group (1631 steps), whereas a decrease was observed in the control group (–386 steps). The baseline values in the two groups were 4365 and 5138 steps/day, respectively. In the baseline evaluation of the EVIDENT II study, the mean number of steps/day as determined with the accelerometer was 9992 in the app+counseling group and 9708 in the counseling only group. This was followed by a decrease in both groups after 3 months, possibly because the baseline values were very high. We have found no studies involving accelerometer interventions in adults with the published data being limited to younger participants. Direito et al [18] compared two intervention groups using two different apps with a control group. Physical activity, evaluated with an accelerometer, decreased in both the control group and in one of the intervention groups after 8 weeks coinciding with the findings of the EVIDENT study, which saw practically no changes in the data assessed with the Physical Activity Questionnaire for Adolescents (PAQ-A). The decrease in accelerometer recordings is probably attributable to a Hawthorne effect associated with utilization of the device—the increase in usual activity being more evident at baseline than after 3 months due to a certain loss of effect. This circumstance could limit the usefulness of the accelerometer in evaluating the effect of the interventions, despite the method being objective.

In the EVIDENT trial, nutritional counseling was seen to increase the overall score of adherence to the Mediterranean diet. Counseling was standardized in both groups with all participants receiving an informative leaflet [20]. This type of nutritional counseling has shown improvements in food habits, with a moderate increase in the consumption of fruit, vegetables, and fiber, especially when written materials are supplied in support of counseling [13]. However, the added use of an app did not result in significant differences between the overall groups or subgroups. There is little evidence of the effectiveness of apps in improving food habits and, in general, the results obtained are modest and come from studies with small sample sizes [33].

Nevertheless, the use of new technologies achieved some change in a study of young individuals aged between 18 and 35 years, with a slight increase in vegetable intake and a decrease in the consumption of sugared beverages [31]. Furthermore, a lesser calorie and fat intake was recorded, resulting in increased weight loss [33]. On the other hand, Coughlin et al [33] considered that heterogeneity in the functional characteristics of the different apps makes it more difficult to draw conclusions and to estimate the magnitude of their effect. In turn, Wang et al [34] suggested that effectiveness can be increased by orienting these tools toward personalized needs, such as self-education and the gaining of awareness of personal food intake. In this regard, one of the novelties of the EVIDENT app is the incorporation of weekly notifications on the benefits and characteristics of the consumption of vegetables, fruit, olive oil, fish, and tomato sauce prepared with vegetables and olive oil—all being traditional ingredients of the Mediterranean diet.

### Limitations

Our study also has several limitations. The nature of the intervention precludes blinding of the participants and this could influence the results obtained. Also, the main findings of the study are based on self-reported information for adherence to both the Mediterranean diet and to PA. Lastly, the recorded loss rate of close to 10% may have biased the study sample composition to some extent because certain populations may have experienced difficulties using the app and consequently decided to leave the study. During the study period, although we recommended not using other apps or wearables that register nutrition and PA, we have no total guarantee that any participant did not use them.

### Conclusions

Physical activity, evaluated with the 7-day PAR, increased more in the app+counseling group than the counseling only group for leisure-time MVPA, although no difference was found when comparing the increase between the two groups. Improved adherence to the app appears to be associated with better results in terms of PA evaluated with the accelerometer. Counseling accompanied by printed materials appears to be effective in improving adherence to the Mediterranean diet, although the app for mobile phones did not increase effectiveness. Further studies are needed to determine which population subgroups may benefit most from interventions based on information and communication technologies.

## Multimedia Appendix 1

Baseline adherence to the Mediterranean diet.

## Multimedia Appendix 2

Subgroup analyses by age, gender, baseline physical activity and weight.

## Multimedia Appendix 3

Subgroup analyses by sociodemographic variables.

## Multimedia Appendix 4

7-day PAR in university women subgroup.

## Abbreviations

7-day PAR: 7-day Physical Activity Recall
MEDAS: Mediterranean Diet Adherence Screener
MET: metabolic equivalent
MVPA: moderate-to-vigorous physical activity
PA: physical activity
