# Supplementary material for: Short-Term Effectiveness of a Mobile Phone App for Increasing Physical Activity and Adherence to the Mediterranean Diet in Primary Care: A Randomized Controlled Trial (EVIDENT II Study)
Source: J Med Internet Res. 2016 Dec 19;18(12):e331. doi: 10.2196/jmir.6814 (PMC5206481; doi:10.2196/jmir.6814)
Supplement: Supplementary file 1 [file jmir_v18i12e331_app1.pdf]

Baseline adherence to the Mediterranean diet

	APPG (415; 49.8%)		CG (418;50,2%)		
**Criteria mediterranean diet**	N	(%)	N	(%)	*P*
1. Using olive oil as the principal source of fat for cooking	389	(94)	394	(94)	.77
2. ≥4 T (1 T=13.5 g) of olive oil/d (eg, used in frying, salads, meals eaten away from home)	154	(37)	135	(32)	.15
3. 2 or more servings of vegetables/d	166	(40)	151	(36)	.25
4. 3 or more pieces of fruit/d	180	(43)	177	(42)	.78
5. 1 serving of red meat or sausages/d	347	(84)	355	(85)	.63
6. 1 serving of animal fat/d	378	(91)	379	(91)	.90
7. 1 cup (1 cup=100 mL) of sugar-sweetened beverages/d	356	(86)	363	(87)	.69
8. ≥7 servings of red wine/week	79	(19)	70	(17)	.42
9. ≥3 servings of legumes/week	98	(24)	81	(19)	.15
10. ≥3 servings of fish/week	163	(39)	183	(44)	.21
11. <2 commercial pastries/week	206	(50)	195	(47)	.41
12. ≥3 servings of nuts/week	149	(36)	123	(29)	.05
13. Preferring white meat over red meat?	282	(68)	261	(62)	.11
14. ≥2 servings/wk of a dish with a traditional sauce of tomatoes, garlic, onion, or leeks sautéed in olive oil	220	(53)	223	(53)	.94
Study participants with a total score ≥ 9 points (n,%)	142	(34)	119	(28)	.09
Score for adherence to Mediterranean Diet (mean± SD)	7.6	2.1	7.4	2.0	.09

APPG: Counseling+APP group, CG: Counseling group. APP: Smartphone application. Categorical variables are expressed as number (n) and (%) and continuous variables as mean ± standard deviation (SD). *P*: statistically significant differences ($P < .05$). ANCOVA test adjusted by baseline measure and Fisher test.
