# Supplementary material for: Short-Term Effectiveness of a Mobile Phone App for Increasing Physical Activity and Adherence to the Mediterranean Diet in Primary Care: A Randomized Controlled Trial (EVIDENT II Study)
Source: J Med Internet Res. 2016 Dec 19;18(12):e331. doi: 10.2196/jmir.6814 (PMC5206481; doi:10.2196/jmir.6814)
Supplement: Supplementary file 2 [file jmir_v18i12e331_app2.pdf]

**Subgroup analyses by age, gender, baseline physical activity and weight**

| **Gender** | | **Mean Difference (APPG-CG)** | **CI 95%** | | ***P*** |
|---|---|---|---|---|---|
| Women | METS minute/week in leisure time | 130.3 | (-38.0 to | 298.6) | .129 |
| | Total MVPA minute /week | 1.9 | (-29.4 to | 33.2) | .905 |
| | Score for adherence to Mediterranean Diet | -0.1 | (-0.4 to | 0.2) | .464 |
| Men | METS minute/week in leisure time | -80.0 | (-330.3 to | 170.4) | .530 |
| | Total MVPA minute /week | -50.9 | (-99.7 to | -2.0) | **.042** |
| | Score for adherence to Mediterranean Diet | 0.1 | (-0.3 to | 0.5) | .529 |
| **Age groups** | | | | | |
| < 40 years | METS minute/week in leisure time | 1.3 | (-271.8 to | 274.4) | .993 |
| | Total MVPA minute /week | -20.6 | (-73.9 to | 32.8) | 0.45 |
| | Score for adherence to Mediterranean Diet | -0.2 | (-0.6 to | 0.3) | 0.49 |
| 40-60 years | METS minute/week in leisure time | 125.8 | (-61.9 to | 313.5) | 0.19 |
| | Total MVPA minute /week | -5.8 | (-45.8 to | 34.2) | 0.78 |
| | Score for adherence to Mediterranean Diet | 0.1 | (-0.2 to | 0.5) | 0.46 |
| > 60 years | METS minute/week in leisure time | 33.1 | (-265.1 to | 331.3) | 0.83 |
| | Total MVPA minute /week | -33.7 | (-83.0 to | 15.6) | 0.18 |
| | Score for adherence to Mediterranean Diet | -0.1 | (-0.6 to | 0.3) | 0.53 |
| **Physical activity** | | | | | |
| Sedentary | METS minute/week in leisure time | 48.1 | (-84.2 to | 180.4) | 0.48 |
| | Total MVPA minute /week | -9.8 | (-39.9 to | 20.4) | 0.53 |
| | Score for adherence to Mediterranean Diet | 0.0 | (-0.3 to | 0.3) | 0.88 |
| Active | METS minute/week in leisure time | 61.2 | (-301.1 to | 423.5) | 0.74 |
| | Total MVPA minute /week | -31.8 | (-89.0 to | 25.5) | 0.28 |
| | Score for adherence to Mediterranean Diet | -0.1 | (-0.6 to | 0.3) | 0.62 |
| **BMI** | | | | | |
| BMI<25 | METS minute/week in leisure time | 53.5 | (-206.6 to | 313.6) | 0.69 |
| | Total MVPA minute /week | -41.8 | (-90.2 to | 6.6) | 0.09 |
| | Score for adherence to Mediterranean Diet | 0.1 | (-0.4 to | 0.5) | 0.81 |
| BMI 25-30 | METS minute/week in leisure time | 92.2 | (-128.8 to | 313.1) | 0.41 |
| | Total MVPA minute /week | 2.9 | (-41.0 to | 46.8) | 0.90 |
| | Score for adherence to Mediterranean Diet | 0.0 | (-0.4 to | 0.3) | 0.84 |
| BMI > 30 | METS minute/week in leisure time | -14.5 | (-262.5 to | 233.5) | 0.91 |
| | Total MVPA minute /week | -19.7 | (-67.8 to | 28.3) | 0.42 |
| | Score for adherence to Mediterranean Diet | -0.1 | (-0.5 to | 0.4) | 0.81 |

APPG: Counseling+APP group, CG: Counseling group. BMI: Body mass index. MVPA: Moderate Vigorous/very vigorous physical activity. METS: metabolic equivalents. *P*: statistically significant differences ($P < .05$). ANCOVA test adjusted by baseline measure comparing changes between intervention and control groups.
