# Supplementary material for: Short-Term Effectiveness of a Mobile Phone App for Increasing Physical Activity and Adherence to the Mediterranean Diet in Primary Care: A Randomized Controlled Trial (EVIDENT II Study)
Source: J Med Internet Res. 2016 Dec 19;18(12):e331. doi: 10.2196/jmir.6814 (PMC5206481; doi:10.2196/jmir.6814)
Supplement: Supplementary file 3 [file jmir_v18i12e331_app3.pdf]

Subgroup analyses by sociodemographic variables

**Civil status**		**Mean Difference (APPG-CG)**	**CI 95%**			***P***
Single	METS minute/week in leisure time	42.1	(-331.8	to	416.0)	0.82
	Total MVPA minute /week	-48.0	(-121.0	to	25.0)	0.20
	Score for adherence to Mediterranean Diet	0.1	(-0.5	to	0.7)	0.74
Married	METS minute/week in leisure time	37.7	(-127.5	to	202.9)	0.65
	Total MVPA minute /week	-7.5	(-38.9	to	24.0)	0.64
	Score for adherence to Mediterranean Diet	0.0	(-0.3	to	0.3)	0.94
Separate	METS minute/week in leisure time	100.1	(-388.8	to	589.0)	0.68
	Total MVPA minute /week	12.3	(-80.2	to	104.8)	0.79
	Score for adherence to Mediterranean Diet	-0.7	(-1.7	to	0.2)	0.14
Widower	METS minute/week in leisure time	300.3	(-797.4	to	1398.0)	0.57
	Total MVPA minute /week	-121.0	(-273.8	to	31.8)	0.11
	Score for adherence to Mediterranean Diet	-0.1	(-1.7	to	1.5)	0.91
**Educational level completed**						
University studies	METS minute/week in leisure time	67.1	(-208.8	to	343.0)	0.63
	Total MVPA minute /week	-1.2	(-52.4	to	50.1)	0.96
	Score for adherence to Mediterranean Diet	0.3	(-0.2	to	0.7)	0.24
Middle or High school	METS minute/week in leisure time	76.6	(-128.3	to	281.6)	0.46
	Total MVPA minute /week	-18.2	(-55.2	to	18.7)	0.33
	Score for adherence to Mediterranean Diet	-0.2	(-0.5	to	0.1)	0.19
Elementary school	METS minute/week in leisure time	-55.7	(-323.0	to	211.5)	0.68
	Total MVPA minute /week	-39.1	(-103.5	to	25.4)	0.23
	Score for adherence to Mediterranean Diet	0.1	(-0.4	to	0.6)	0.74
**Work situation**						
Works outside of home	METS minute/week in leisure time	95.1	(-85.7	to	275.8)	0.30
	Total MVPA minute /week	-22.4	(-60.1	to	15.2)	0.24
	Score for adherence to Mediterranean Diet	0.1	(-0.3	to	0.4)	0.74
Homemaker	METS minute/week in leisure time	55.0	(-311.7	to	421.8	0.77
	Total MVPA minute /week	-2.6	(-70.2	to	64.9)	0.94
	Score for adherence to Mediterranean Diet	0.0	(-0.5	to	0.5)	0.93
Retired	METS minute/week in leisure time	20.0	(-337.1	to	377.1	0.91
	Total MVPA minute /week	-15.8	(-77.3	to	45.7)	0.61
	Score for adherence to Mediterranean Diet	-0.2	(-0.7	to	0.3)	0.49
Student	METS minute/week in leisure time	744.4	(-632.8	to	2121.6)	0.26
	Total MVPA minute /week	-7.5	(-191.7	to	176.6)	0.93
	Score for adherence to Mediterranean Diet	0.0	(-3.0	to	3.0)	0.99
Unemployed	METS minute/week in leisure time	63.8	(-327.3	to	454.9)	0.75
	Total MVPA minute /week	-13.9	(-107.8	to	80.0)	0.77
	Score for adherence to Mediterranean Diet	-0.1	(-0.8	to	0.6)	0.76

APPG: Counseling+APP group, CG: Counseling group. BMI: Body mass index. MVPA: Moderate Vigorous/very vigorous physical activity. METS: metabolic equivalents. *P*: statistically significant differences ($P < .05$). ANCOVA test adjusted by baseline measure comparing changes between intervention and control groups.
