# Supplementary material for: Short-Term Effectiveness of a Mobile Phone App for Increasing Physical Activity and Adherence to the Mediterranean Diet in Primary Care: A Randomized Controlled Trial (EVIDENT II Study)
Source: J Med Internet Res. 2016 Dec 19;18(12):e331. doi: 10.2196/jmir.6814 (PMC5206481; doi:10.2196/jmir.6814)
Supplement: Supplementary file 4 [file jmir_v18i12e331_app4.pdf]

7-day PAR in university women subgroup

**N= 144** (APPG=67, CG=77)	**Unadjusted**		**Adjusted**	
	Mean Difference (APPG-CG) CI 95%	*P*	Mean Difference (APPG-CG) CI 95%	*P*
Total minutes moderate activity	67.5 (-19.0 to 153.9)	.12	37.3 (-33.3 to 107.8)	.29
Total minutes moderate activity in leisure time	75.4 (2.3 to 148.5)	**.04**	50.5 (-18.2 to 119.2)	.15
Total minutes vigorous/very vigorous activity	-2.8 (-33.5 to 27.9)	.86	4.7 (-25.1 to 34.5)	.76
Total minutes vigorous/very vigorous activity in leisure time	1.0 (-29.4 to 31.4)	.95	9.8 (-19.1 to 38.7)	.50
Total minutes moderate vigorous/very vigorous activity	64.7 (-19.3 to 148.6)	.13	47.9 (-24.7 to 120.5)	.19
Total minutes moderate vigorous/very v. activity in leisure time	76.4 (-2.4 to 155.1)	.06	57.9 (-15.9 to 131.7)	.12
METS minute/week	260.3 (-100.4 to 621.0)	.16	210.2 (-113.3 to 533.7)	.20
METS minute/week in leisure time	323.4 (-28.2 to 675.1)	.07	258.3 (-70.5 to 587.0)	.12

7-day PAR **:** 7-day physical activity recall questionnaire. APPG: Counseling+APP group, CG: Counseling group. METS: metabolic equivalents.
P: statistically significant differences (*P* < .05). ANCOVA test unadjusted and adjusted by baseline measure.
